# Lessons Learned From Integrating Infant and Young Child Feeding Counseling and Iron-Folic Acid Distribution Into Routine Immunization Services in Ethiopia

**DOI:** 10.9745/GHSP-D-22-00166

**Published:** 2022-10-31

**Authors:** Natasha Kanagat, Adriana Almiñana, Belayneh Dagnew, Lisa Oot, Amare Bayeh, Daniel Girma, Tewodros Alemayehu, Getu Molla Tarekegn, Yohannes Lakew Tefera, Meseret Zelalem Tadesse, Hiwot Darsene Dimd, Zenaw Adam

**Affiliations:** a JSI Research & Training Institute, Inc., Arlington, VA, USA.; b JSI Research & Training Institute, Inc., Addis Ababa, Ethiopia.; c Federal Ministry of Health, Addis Ababa, Ethiopia.

## Abstract

Immunization programs reach more children and communities than any other health intervention, thus making immunization a promising platform for integrating other essential health services. There is a dearth of literature on integrating nutrition interventions, such as infant and young child feeding (IYCF) counseling and iron-folic acid (IFA) supplementation, into routine immunization services.

To address this evidence gap, a 15-month pilot study (August 2019 to November 2020) tested the feasibility of integrating IYCF counseling and IFA supplement distribution into immunization service delivery in Ethiopia. The interventions focused on joint microplanning for integrated services (including estimating target populations for all services), revising client flows for service delivery, and providing on-the-job support to HWs for implementing and monitoring integrated service delivery.

Findings suggest that planning for and delivering IYCF counseling and IFA supplementation with immunization services is feasible. Integrating these services provided opportunities for collaborative planning and enabled health workers (HWs) to offer multiple services to clients through 1 interaction. However, HWs felt that additional human resources were needed to manage integrated services, especially during integrated outreach and mobile service delivery. HWs also reported that communities appreciated accessing 2 services in 1 visit but expressed reservations about the longer wait times.

Countries may consider expanding fixed and outreach immunization services to provide integrated service delivery provided that it is feasible, sustainable, of high quality, and incorporates the careful planning, follow-up, and increased human and financial resources needed to reinforce new practices and expand access to a broader array of health services.

## Abstract (precis)

This study’s findings demonstrate that integrating counseling on infant and young child feeding and iron-folic acid supplement distribution into immunization services is achievable and can promote greater accessibility to other health services in immunization and beyond.

## Teaser

**Key Findings:**

- Integrated planning helped to maintain focus on both programs; infant and young child feeding (IYCF) counseling was done more regularly.
- The service delivery experience was mixed. Health workers (HWs) initially struggled with the revised workflow, heavier workload, and data collection responsibilities. After receiving sustained technical assistance, they noted that their planning, reporting, and service delivery improved.
- HWs noted that providing integrated services was easier during facility-based sessions than outreach or mobile services due to the increased need for human resources, supplies, and data recording.

**Key Implications:**

- For countries seeking to integrate additional services into immunization, we recommend an assessment to gauge feasibility related to health system inputs. Once the decision to offer integrated services is made, the district-level integrated microplan will help map out program requirements. Adjustments necessary for effective integration can be identified during microplanning and strategies to ensure broad engagement of stakeholders, especially community members and technical partners.
- Countries may benefit from initially integrating IYCF counseling into facility-based immunization sessions since mobile and outreach sessions require more planning related to supplies, human resources, and data recording tools.

## INTRODUCTION

For a primary health care system to be people centered and responsive to population needs throughout the life course, it must feature integrated health service delivery.^1^^,^^2^ According to the World Health Organization (WHO), integrated health services are essential if countries want to ensure populations receive timely and high-quality preventative and curative care based on their needs.^3^

Because immunization programs reach more children and communities than any other health intervention^4^ and promote regular contact between communities and the health system, they are a favorable way to deliver additional health services. Conversely, we know that if households do not receive immunization services, they are likely to forgo other primary health care services.^5^

Recognizing the potential for improving health coverage and equity, global immunization strategies like Immunization Agenda 2030,^6^ Gavi 5.0,^7^ and others^8^^–^^11^ emphasize integrating other health services with immunization. Integrating immunization and nutrition interventions is logical given their complementarity, overlapping timeframes, and the potential to reinforce gains in nutrition and immunization outcomes.^12^

The literature on integrated nutrition and immunization interventions has focused on providing vitamin A supplementation during immunization campaigns and child health weeks. Findings suggest that integrating these interventions improved coverage for both, but authors noted that routine immunization (RI) rather than sporadic campaigns should be used as the platform for integrating nutrition to ensure that the services are provided in an organized, consistent manner.^13^^–^^15^ However, there is a dearth of literature on integrating other nutrition interventions into RI. To address this evidence gap, a 15-month pilot study in Ethiopia tested the feasibility of incorporating counseling on integrated young child feeding (IYCF) and iron and folic acid (IFA) supplement distribution into immunization service delivery. In this article, we describe pilot study learnings related to planning and delivering integrated services, including factors that hindered or enabled implementation, and share recommendations for integrating immunization and nutrition services in other settings. We define key terms used in RI planning and delivery (Box 1).^16^

BOX 1 **Definition of Key Terms Used in Routine Immunization Planning and Delivery^a^**

**Microplan:** A document that defines how to reach clients, how many people should be targeted for services in a given area, and how frequently quality services should be provided. Microplanning involves stakeholders at each level of the health system.

**Static/fixed services:** Services offered in a health facility (on specified days and times).

**Outreach services:** Services delivered in a community by health workers who visit the community and return to the health facility in the course of the working day.

**Mobile services:** Sessions that are conducted by teams of health workers who travel to locations distant from a health facility. The teams usually stay in the community for at least 1 night. Mobile sessions are scheduled as needed, with teams visiting homes, fields, workplaces, and schools, or wherever the population is living.

^a^Note that these terms may be used differently in other health programs. All definitions are from World Health Organization.^16^

Routine immunization rather than sporadic campaigns should be used as the platform for integrating nutrition and immunization to ensure that the services are provided in an organized, consistent manner.

## UNIVERSAL IMMUNIZATION THROUGH IMPROVING FAMILY HEALTH SERVICES PROJECT

Ethiopia has made gains in increasing immunization coverage. From 2000 to 2016, coverage for the first dose of the diphtheria, pertussis, and tetanus (DPT) vaccine increased from 40% to 73% and for the third dose of DPT (DPT3) increased from 18% to 53%.^17^^,^^18^ Nevertheless, the country has not achieved its goal of 85% DPT3 coverage.^19^ Progress in nutrition has been more uneven. Although the prevalence of exclusive breastfeeding (EBF) improved from 55% to 58% from 2000 to 2016, minimum acceptable diet only increased slightly from 4% in 2011^20^ to 7% in 2016, which is extremely low. In 2016, 42% of women took iron tablets, a significant improvement from 17% in 2011. The percentage of women who received 2 or more doses of tetanus toxoid injections during pregnancy changed slightly from 34% to 41% between 2011 and 2016.

For all these indicators, there are significant regional variations. For example, Somali and Afar have geographically remote populations and are consistently underserved, highlighting the importance of not only reaching these populations but doing so through integrated services. Studies in Ethiopia indicate that women who have contact with HWs at health facilities (HFs) have more favorable practices related to EBF, complementary feeding, and IFA than women who lack this contact.^17^^,^^18^^,^^21^

In support of Ethiopia’s efforts to address barriers to equity and reach every child, JSI Research & Training Institute, Inc. (JSI) collaborated with Ethiopia’s Expanded Program on Immunization (EPI) from 2011 to 2021 to implement the project Universal Immunization through Improving Family Health Services (UI-FHS), which aimed to improve the availability, utilization, quality, and sustainability of immunization services (Box 2).

BOX 2 **Reaching Every District and Universal Immunization through Improving Family Health Services Project**

The essential components of Reaching Every District include planning and management of resources, reaching all eligible populations, engaging with the community, providing supportive supervision, and monitoring and using data for action. To bolster implementation of the 5 Reaching Every District components, the Universal Immunization through Improving Family Health Services (UI-FHS) approach used quality improvement tools to enable health personnel to identify local problems in immunization services and design solutions.

In 2019, UI-FHS, Ethiopia’s regional health bureaus, woreda (district) health offices, and nutrition partners began exploring integrating select nutrition services into the immunization platform. Stakeholders prioritized integrating IYCF counseling and IFA supplement distribution into immunization because of the similarities in target populations, the degree to which the services complement each other, and the overlap in the life stage when the interventions are delivered. Table 1 identifies the times when immunization and nutrition-related services could be provided simultaneously.

**TABLE 1.** Time Frames for Proposed Integrated Immunization and Nutrition Services in Ethiopia

**Vaccine(s) Provided**	**Target Time/Age for Intervention**		**Nutrition-Related Services/Guidance to Provide** **(IFA and IYCF)**
Tetanus toxoid	Pregnancy	Pregnancy	IFA supplementation, counseling on nutrition for pregnant and lactating women, recommendation regarding initiating breastfeeding immediately after birth
BCG, OPV0	At birth	0–6 months	Breastfeeding in first hour of life, colostrum feeding, exclusive breastfeeding counseling
First dose: Penta, OPV, PCV, Rota	6 weeks		Exclusive breastfeeding counseling
Second dose: Penta, OPV, PCV, Rota	10 weeks		
Third dose: Penta, OPV, PCV, Rota, IPV	14 weeks		
First dose: Measles	9 months	6–23 months	Continued breastfeeding, introduction of complementary foods, counseling for complementary feeding (messages may vary based on age of child)
Second dose: Measles	15 months		

Abbreviations: BCG, bacille Calmette-Guerin; IFA, iron and folic acid; IYCF, infant and young child feeding; IPV, inactivated polio vaccine; OPV0, oral polio vaccine given at birth; penta, pentavalent vaccine; PCV, pneumococcal vaccine; Rota, rotavirus.

Ethiopia’s immunization program, managed by the Ministry of Health, employs static, outreach, and mobile strategies to deliver immunization sessions to ensure that mothers and children can access services close to where they live and to minimize the burden of traveling to HFs. Tetanus toxoid vaccines are offered to pregnant women, followed by RI of children in the first 15 months of life. In accordance with WHO guidelines,^22^^–^^25^ the Ethiopia National Nutrition Programme^26^ recommends distributing IFA tablets to pregnant women to minimize the risk of complications from anemia and offering IYCF counseling on EBF to pregnant women and mothers with children aged younger than 2 years and on the timely introduction of complementary feeding to mitigate malnutrition. IYCF counseling is generally provided during static sessions with some outreach, while IFA tablets are primarily distributed through static antenatal care (ANC) sessions.

## PILOT STUDY DESIGN

The pilot study was implemented from August 2019 to November 2020 in 47 HFs (all health centers and health posts within each district) (Table 2).

**TABLE 2.** Distribution of Health Facilities by District and Region, Ethiopia

**Region**	**District**	**Health Facilities, No.**
Afar	Assaieta	11
Somali	Adadle	8
Gambela	Godere	9
Benishangul Gumuz	Bullen	19
Total		47

HFs that met the following criteria were selected to participate in the study: HFs with HWs who had conducted integrated static and mobile or outreach services in the 3 months before data collection (to ensure that HWs were familiar with the activity); inclusion of rural HFs and 1 urban HF per district where possible; and HFs close to the district capital so that data collection could be completed in 1 week.

During the pilot study, project staff implemented the following activities.

- Facilitated advocacy and orientation meetings with district and regional staff and established a technical working group that engaged regional staff and implementing partners to monitor data and conduct supportive supervision.
- Adapted the microplanning tool for integrated services and provided integrated microplanning training on planning joint immunization and nutrition service delivery, including estimating target populations for each service. HWs were trained on how to provide nutrition and IYCF services including counseling.
- Revised client flows for service delivery (Figure) to ensure that HWs understood how to provide integrated services in a standardized manner.

**FIGURE:**
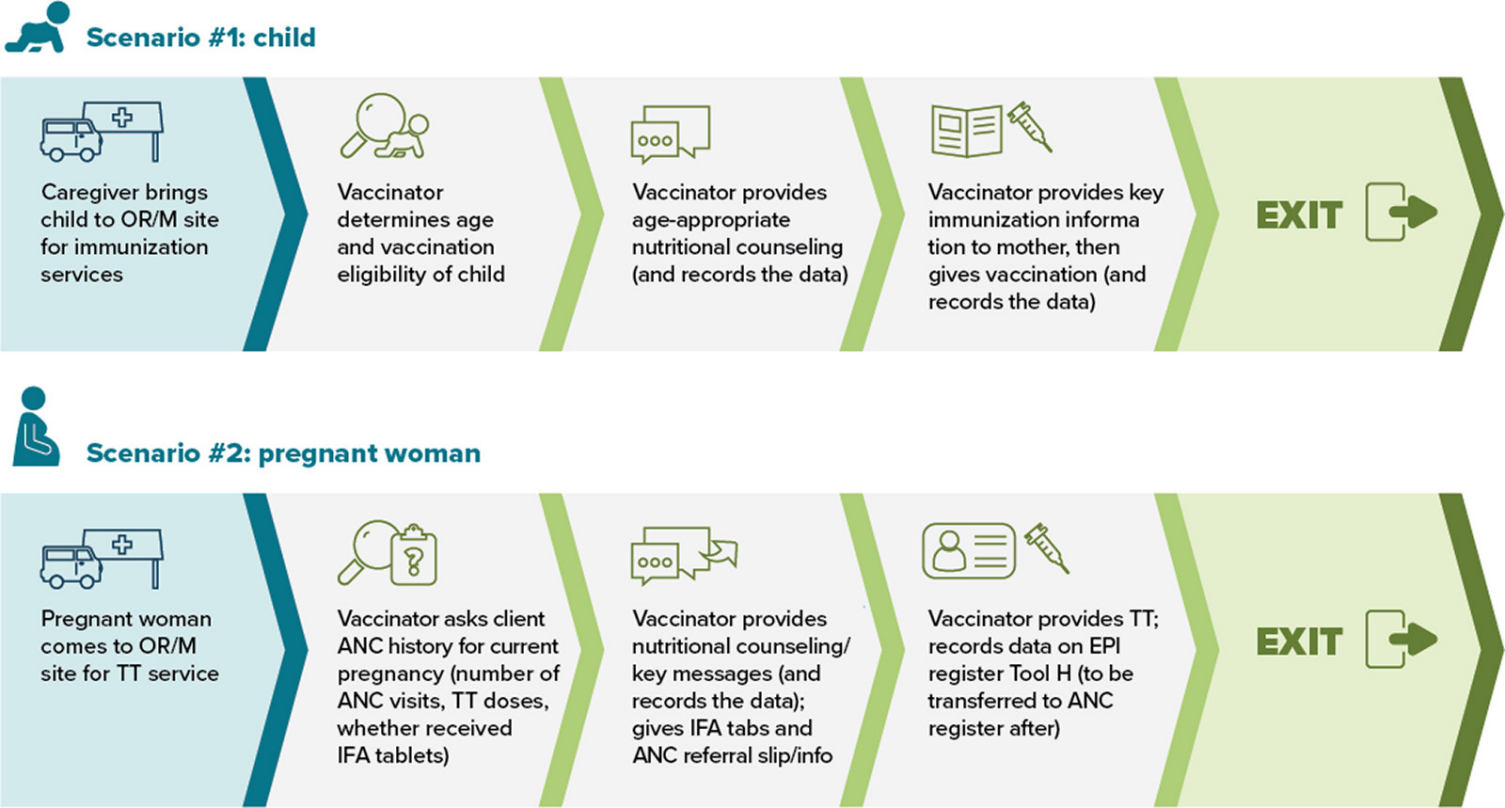
Proposed Client Flow Graphic for Integrated Nutrition and Immunization Outreach and Mobile Sessions in Ethiopia^a^ Abbreviations: ANC, antenatal care; IFA, iron and folic acid; OR/M, outreach/mobile; TT, tetanus toxoid. ^a^Similar client flow graphics were proposed for static sessions in health facilities.
- Used supportive supervision and on-the-job support to provide technical assistance to HWs around providing integrated services as per the plan, supported them in using data recording tools, and addressed their concerns.
- Supported the monitoring of integrated services using adapted data recording tools. Since IYCF is not tracked in the administrative data system, the project modified the immunization tally sheet by creating space to tally IYCF and/or IFA. It also developed a modified form for gathering basic information on pregnant women that was copied over to the ANC register following integrated sessions, allowing HWs to continue to carry just the immunization register to mobile and outreach sessions. During implementation, monitoring data were examined to understand if integrated services were being provided and to address concerns raised by HWs related to service provision or recording data.
- Encouraged existing quality improvement teams, which typically focused on problem-solving for immunization service delivery, to expand their focus to include nutrition services.
- Provided guidance around HW roles and responsibilities during the provision of integrated outreach and mobile services. HF staff took the lead in deciding on the distribution of roles based on the guidance and their reality. In most regions in Ethiopia, health extension workers are qualified and able to administer vaccinations. In some pastoralist areas, they are not qualified and thus do not directly administer vaccines (other cadres do). Even in these pastoralist areas where health extension workers can't vaccinate, they support the delivery of immunization services through various means, like assisting with data recording during sessions, tracing defaulter children, or engaging and mobilizing communities to attend outreach.

Project staff provided guidance around how HW roles and responsibilities may change during the provision of integrated outreach and mobile services.

Although the project did not supply IYCF materials or IFA tablets, the project staff discussed the importance of ensuring the supply of all the materials during microplanning and emphasized this in the microplan template. In Gambela, there were initial stock-outs of IFA tabs at the health post level (lowest level facility) because ANC was not being delivered to health posts, as is usually expected per national guidelines. This prompted discussions about ensuring IFA tab availability at health posts, and the services were then restored in line with national guidelines.

### Study Rationale and Methodology

The pilot study used a qualitative descriptive design to answer the following questions.

1. How did integrated planning influence planning for immunization and nutrition services?
  - What was the experience of respondents at regional, district, and facility levels?
  - Which aspects of integrated planning worked well and which presented challenges?
2. How did integrated service delivery influence immunization and nutrition service delivery?
  - What was the experience of respondents at regional, district, and facility levels?
  - Which aspects of integrated service delivery worked well and which presented challenges?

### Data Collection, Management, and Analysis

Data collection methods included key informant interviews (KIIs), observations, and document review.

1. KIIs with HF HWs, regional health bureaus nutrition officers and EPI focal persons, woreda health office EPI focal persons, nutrition partners, and immunization specialists from UI-FHS. The KIIs were designed to elicit respondents’ experiences and perceptions related to integrating IYCF counseling and IFA supplementation into immunization services. Key informants were selected to capture the perspectives of those responsible for managing the program and those with direct service delivery experience who could share insights into community perceptions of integrated services.
2. Observations of integrated sessions to document whether HWs provided integrated services and completed data collection forms in accordance with the guideline.
3. Document reviews to verify the availability of integrated microplans; up-to-date session plans for static, outreach, and mobile immunization sessions; and session tally sheets. Session observations and document reviews were used to triangulate learnings from the KIIs.

From December 2020 to January 2021, the team conducted 43 KIIs, observed 4 integrated sessions, and completed document reviews at 17 HFs. Document review data were not available from 5 health facilities in Benishangul-Gumuz due to security concerns at the time of data collection that prevented site visits; KIIs for these facilities were conducted over the phone. To avoid conflict of interest and bias during data collection, staff involved in implementing the pilot were not members of UI-FHS data collection teams. Four researchers with advanced degrees in public health, expertise in qualitative research, and fluency in Amharic and Somali conducted data collection. They underwent a 2-day training on study objectives, the project’s background, qualitative research, data collection tools, and immunization and nutrition programming in Ethiopia. Respondents gave their written informed consent to participate. Data collectors used interview guides and audio-recorded interviews. The audio files were transcribed in English and transcripts were imported into NVivo 12 for coding and analysis. An inductive content and thematic analysis approach was used to identify themes related to the main research questions. Team members divided the transcripts and independently coded interviews. Kappa coefficient scores were compared to gauge the consistency of coding. Researchers discussed scores that fell below 80 and recoded the data based on consensus. To triangulate data from the KIIs, the team analyzed Excel data from document reviews and observations.

### Ethical Approval

The JSI Institutional Review Board (IRB) (IRB #20-51E) determined that the study was exempt based on the U.S. Code of Federal Regulations CFR 46.101 (b) (2), which covers study activities without identifiers or sensitive questions that could result in harm. No participants in the study were aged younger than 18 years. All of the regional health bureaus approved the implementation of this study.

## THEMATIC FINDINGS

Overall findings from the pilot suggest that planning for and delivering IYCF counseling and IFA supplementation with immunization services was feasible. Integrating these services provided opportunities for collaborative planning and enabled providing multiple services to clients through 1 interaction. However, HWs felt that additional human resources were needed to manage integrated services and reduce the burden on already overworked staff, especially regarding integrated outreach and mobile service delivery. We present key themes that emerged from our analysis.

### Collaborative Planning and Target Setting

Microplanning for integrating IYCF counseling and IFA supplementation and immunization programs requires mapping where communities are located and determining how best to reach populations through shared resources. Respondents explained that before the pilot, nutrition and immunization activities were planned independently by each program, resulting in siloed and parallel planning and service delivery systems.

Key informants noted that microplans were developed by the woreda health office, HFs, community members, and immunization and nutrition partners using a participatory approach, which resulted in the incorporation of diverse views in planning. The HFs used the microplans to develop plans for static, outreach, and mobile immunization sessions that incorporated counseling for IYCF and distribution of IFA. Because respondents considered integrated microplanning more efficient and cost effective than planning for immunization and nutrition services separately, they suggested integrating additional interventions into the microplanning process. Respondents from Afar specifically shared an interest in integrating family planning services to expand the reach of those services. The document review revealed that 16 of the 17 HFs had a copy of the integrated microplans and session plans.

*The integrated plan makes work easier because 2 things [are] done at single time.* —HW, Somali

Respondents considered integrated microplanning more efficient and cost effective than planning for immunization and nutrition services separately and suggested integrating additional interventions into the microplanning process.

Fifteen microplans included target numbers for all population categories (pregnant women, children aged 0–6 months, and children aged 6–23 months) to be reached with integrated services. Although some key informants did not experience challenges setting targets during microplanning, others reported difficulty setting targets for large nomadic populations, which resulted in a mismatch between the actual number of children who attended mobile and outreach sessions and the number of children HWs had estimated in microplans. Respondents recommended updating microplans every month to keep pace with changes in catchment area populations. Notably, challenges with establishing accurate targets are not unique to integrated microplanning but common in immunization microplanning.

*The challenge is mainly the initial data gathering at those hard to reach areas to come up with clear eligible target counting.* —EPI Officer, Benishangul-Gumuz

### Managing Supplies, Clients, and Reporting

The pilot study assessed changes in workflow, reporting, and supply management that resulted from integrating immunization and nutrition services. Combining IYCF and IFA with immunization services required HWs to reorganize their service delivery workflow. For example, they needed to adjust the sequence in which services were offered to optimize the flow of clients and deliver all required services without compromising quality. All of the HWs reported that they offered IYCF counseling before vaccinating because when caregivers focus on soothing a child who is crying after immunization, they were less able to pay attention to nutrition counseling. HWs noted that when integrated sessions got busy, they required additional HWs to provide services. In these situations, respondents recommended training community members or volunteers to conduct counseling to enable HWs and nurses to focus on immunizations.

KIIs with HWs and session observations suggest that HWs determined whether to offer a mother individual or group IYCF counseling based on the number of clients present, the amount of time available to complete tasks, the number of HWs present to complete the tasks, and the child’s weight. HWs were trained to offer individual counseling but often provided group counseling, resulting in inconsistent service quality. However, in all 4 observed sessions, HWs consistently communicated the 5 key immunization messages. Of the 4 sessions, 2 sessions included women eligible for IYCF counseling which they received as planned.

In addition to service considerations, integrating immunization and nutrition services requires effective management of multiple supplies—vaccine carriers, scales, counseling cards, IFA tablets, vaccines, immunization registers, and data recording forms. HWs reported that it was easier to conduct integrated services at static sites than through outreach and mobile services because all of the necessary supplies were present in the HFs. HWs noted that carrying supplies to outreach sessions was challenging, but community volunteers often helped them.

*We do go for outreach and carry many things, including the registration books, all required supplies, safety box. [And] it is not easy to carry all these things since we don’t have a vehicle to drop us at the outreach site.* —HW, Somali

HWs reported that it was easier to conduct integrated services at static sites than through outreach and mobile services because all of the necessary supplies were present in the HFs.

HWs also stated that while integrated outreach sessions took longer, they did not have to make separate trips to a community to provide nutrition and immunization services. Overall, they found it beneficial to reach communities with both services in 1 visit.

*Through the integrated work we saved time and energy because instead of going 2 times to deliver immunization and nutrition services, we just do it once. It is also kind of waste of time if you travel a long distance and only provide immunization services and come back without providing nutrition services to the children and pregnant women.* —HW, Somali

While HWs requested per diems and transport for outreach and mobile services and funds to cover these costs were typically included in microplans, the funds were not consistently provided to HWs. This is a systemic challenge that is not unique to integrated services.

HWs were tasked with completing the reporting requirements for all services provided during integrated sessions. In 2 of the 4 observed sessions, HWs did not complete the integrated reporting forms during the session. Instead, they recorded immunization service data in the EPI register and tally sheet and made informal notes about specific nutrition services, intending to complete the integrated reports after the session. The team could not confirm whether HWs followed through on this plan.

*The integration requires more of a teamwork to register, screen, and counsel, provide vaccine, IFA for pregnant mothers, and tally services offered. Therefore, it requires more human resource for effective session. This is mostly true for outreach and mobile sites. [We] don’t have a problem at static one.* —EPI focal person, Benishangul-Gumuz

### Importance of Supportive Supervision and Mentorship

The HWs interviewed expressed appreciation for the supportive supervision they received during the early months of the pilot. During these visits, HWs learned to plan for and manage integration activities and complete the integration-related reporting forms. HWs recommended that these visits be scheduled more frequently, a minimum of once monthly. Of 17 HFs, 15 received supportive supervision visits focused on integration during the time of the pilot study.

*We were making mistakes while completing the format because we did not understand it well… [woreda health office and JSI] showed us all the things in the format on the spot during the static session. It has not been a challenge once we have understood it well.* —HW, Gambela

### Strengthened Linkages Between Services and Increased Reach of Nutrition Services

Integration strengthened linkages between nutrition and immunization services. Before this activity, key informants explained that IYCF counseling, IFA tablet distribution, and immunization operated as siloed services, and counseling on EBF and complementary feeding were provided in an ad hoc manner. By integrating the services, HWs reported that they were more organized and consistent in providing IYCF counseling to all clients.

*We used to teach mothers about exclusive breastfeeding and complementary feeding. However, it was not regular like this, but it was only provided when mothers came to health posts.* —HW, Afar

HWs noted that when a child presented for immunization services, they offered IYCF counseling, and when a pregnant woman sought immunization services, such as tetanus toxoid immunization, they offered IFA tablets and encouraged completion of all ANC visits. Women who gave birth at home were advised to complete their child’s immunization schedule and counseled on IYCF.

*The integrated approach helps to provide iron for pregnant women who do not attend ANC services, and for the immunization program, it helps to find newborns that are born at home.* —HW, Afar

In addition to providing immunization and nutrition services, some HWs screened children for malnutrition. Children identified as malnourished during integrated outreach sessions were referred to the Outpatient Therapeutic Programme. In some instances, HWs who managed Outpatient Therapeutic Programme services also checked the immunization status of children and referred unvaccinated children to an integrated outreach session or static clinic. HWs reported that these connections between nutrition and immunization services did not occur before integration. It is worth noting that the intervention focused on IYCF and IFA; screening for malnutrition was an unexpected adaptation that some HWs incorporated organically into their work.

### Mixed Community Response

During KIIs, HWs were asked for their perceptions of community responses to providing integrated services. HWs indicated that the community response was mixed. Community members reported to HWs that they preferred integrated services because they enabled them to access multiple services in a single visit. However, HWs also reported that they received several complaints about integrated sessions taking too long and that some communities felt that IYCF counseling was not critical. Communities reported a preference for outreach sessions (where services were brought to them) over static service delivery. HWs also reported that they perceived an overall increase in community awareness about immunization and nutrition services because each service reinforced the other. For example, pregnant women began to request IFA tablets.

*First of all, the EPI and nutrition services and I have seen significant changes. [These] include mothers who come to the health post to receive vaccination and nutrition services, and the mothers kept their appointments at the health post, and understanding of IFA tabs and its benefits for pregnant women, and [pregnant] mothers come to us and ask for IFA tabs, which means that the understanding of the community is very good.* —HW, Somali

*Some of them find it interesting but many of them say, “Let me go, I have to return and cook food for the family, I have long walk ahead…” So they don’t want to stay long, rather they ask for another appointment.* —HW, Afar

HWs also reported that the engagement of community members was essential for integrated service uptake. Quality improvement teams composed of HWs and community members supported integration by communicating the time and location of integrated outreach sessions. Of 17 facilities, 13 reported having a quality improvement team that discussed integrated nutrition and immunization services.

*Every work we do with the kebele [community] leader, the community volunteers and other community are part of it and we cannot [do] anything without involving them… They are the one[s] who are close to the community and they [know] the best way to deal with the community.* —HW, Somali

HWs also reported that the engagement of community members was essential for integrated service uptake.

## DISCUSSION

This study contributed to the knowledge base related to the planning and delivery of IYCF counseling and IFA supplementation with RI services. Findings from the pilot study suggest that integration was feasible, especially during the planning stages, because it seemed to result in better planning for both services when compared to planning these services through separate vertical programs. Findings related to the feasibility of codelivering the services through static, outreach, and mobile were mixed.

Integrated microplanning facilitated a bottom-up approach to identifying eligible women and children for nutrition and immunization services and provided an opportunity to discuss adjustments needed for service delivery mechanisms, workflows, community engagement, and re-sourcing. The challenges with target setting are not specific to integration but are systemic; efforts are underway at the global level to address these issues.^27^^,^^28^

Regarding service delivery, HWs who participated in the pilot viewed integration favorably but reported challenges related to increased workloads, burden of carrying additional supplies to outreach and mobile sites, and time to learn to use new data recording tools. HWs observed that community members appreciated having multiple health services brought to them but also felt burdened by increased wait times. This finding has been echoed in other studies.^22^^,^^25^^,^^29^^,^^30^ Wallace et al. observed the amount of time needed to deliver 11 maternal and child health interventions in Cameroon, Ethiopia, and Mali and found that immunizations require less time compared to other interventions.^31^ They emphasized that “adding more services without additional HWs might increase patient waiting times, resulting in dissatisfaction and perceived poor quality of care, as well as decreased health care utilization by mothers.” Thus, it will be important for other intervention designers and implementers to consider community perceptions and address their concerns to ensure successful outcomes.

HWs also emphasized the role of supervision, mentorship, and coaching in increasing their confidence in providing integrated services, as well as the importance of community engagement to raise awareness about the services and address their concerns. These findings are not specific to integration, are common in health systems research broadly, and have been documented in multiple studies, highlighting the importance to HWs of supervision and community support in planning, service delivery, and troubleshooting obstacles.^31^^–^^35^

HWs also emphasized the role of supervision, mentorship, and coaching in increasing their confidence in providing integrated services.

Although this study provides insights into planning and delivering integrated services, we call for additional studies to advance understanding of how best to codeliver these interventions in different contexts using varied strategies (i.e., via static, outreach, and mobile service delivery). Salam et al.^36^ stated the need to examine how integrating nutrition and other interventions into immunization can improve the reach and coverage of both interventions and address gaps related to equity and gender. As more vaccines enter the market, the interest in codelivering services will increase. For example, a recent WHO position paper on the malaria vaccine also explores the potential for coadministering the malaria vaccine with childhood immunizations and other preventative interventions to maximize reach and coverage.^37^

Finally, since this study assessed the quality of service delivery through informal observation of a limited number of integrated sessions systematic research is required to examine the extent to which quality is maintained when services are codelivered.

### Limitations

This study had 2 important limitations. First, rather than scheduling observations of integrated sessions, we only observed sessions that occurred while the project team was conducting KIIs or document reviews. As a result, we observed a limited sample of 4 sessions, which offered some insights into service quality but were not able to conduct a broader examination of implementation and quality issues in integrated services. Second, we intended to incorporate monitoring data to examine the process of integration and coverage of both interventions through analysis of output data, but due to poor data quality, we could not analyze the extent to which services were integrated as well as changes in coverage or uptake of services as we had anticipated. We also note that this pilot study was conducted from August 2019 to November 2020, which overlaps with the coronavirus disease (COVID-19) pandemic. Although we did not specifically seek to understand the effect of the pandemic on service delivery, we can hypothesize that communities and HWs may have had reservations in providing and utilizing health care services.

## CONCLUSIONS

This study highlights the benefits and challenges of integration. Because this was a pilot study, we recommend further research to expand our knowledge and understanding of how best to codeliver IYCF and RI services. Ministries of health should assess the advantages and drawbacks of integrating IYCF and RI taking into consideration the findings from the pilot study and their unique situations. In summary, integrated microplanning is an essential step that requires the broad engagement of all stakeholders, including HWs, community members, and partners. Careful monitoring of implementation, and—for HWs—ongoing support from supervisors, are critical for reinforcing new practices and troubleshooting challenges that may arise, such as when using data recording tools. Sufficient human resources for health are critical for offering integrated services, particularly outreach and mobile services. Finally, community engagement strategies will be important to ensure community acceptance of and utilization of services.

## Notes

**Peer Reviewed**

## References

[1] World Health Organization (WHO). *Integrating Health Services*. WHO; 2018. Accessed October 12, 2022. https://www.who.int/docs/default-source/primary-health-care-conference/linkages.pdf

[2] Clements CJ Nshimirimanda D Gasasira A. Using immunization delivery strategies to accelerate progress in Africa towards achieving the Millennium Development Goals. Vaccine. 2008;26(16):1926–1933. 10.1016/j.vaccine.2008.02.032. 18343540

[3] Service organizations and integration. World Health Organization. Accessed October 17, 2022. https://www.who.int/teams/integrated-health-services/clinical-services-and-systems/service-organizations-and-integration

[4] Gavi, the Vaccine Alliance. *Immunisation: Strengthening Primary Healthcare for Universal Health Coverage*. Gavi; 2019. Accessed October 12, 2022. https://www.gavi.org/sites/default/files/publications/Immunisation%20-%20a%20platform%20for%20universal%20health%20coverage.pdf

[5] Santos TM Cata-Preta BO Mengistu T Victora CG Hogan DR Barros AJD. Assessing the overlap between immunisation and other essential health interventions in 92 low- and middle-income countries using household surveys: opportunities for expanding immunisation and primary health care. EClinicalMedicine. 2021;42:101196. 10.1016/j.eclinm.2021.101196. 34805814 PMC8585628

[6] World Health Organization (WHO). *Immunization Agenda 2030 (IA2030): A Global Strategy to Leave No One Behind*. WHO; 2020. Accessed October 12, 2022. https://www.who.int/docs/default-source/immunization/strategy/ia2030/ia2030-document-en.pdf

[7] Gavi 5.0: Phase V (2021–2025). Gavi, the Vaccine Alliance. Accessed October 12, 2022. https://www.gavi.org/our-alliance/strategy/phase-5-2021-2025

[8] World Health Organization (WHO). *GIVS Global Immunization Vision and Strategy 2006–2015*. WHO; 2005. Accessed October 12, 2022. https://apps.who.int/iris/bitstream/handle/10665/69146/WHO_IVB_05.05.pdf

[9] World Health Organization (WHO). *Global Vaccine Action Plan Monitoring, Evaluation & Accountability: Secretariat Annual Report 2020*. WHO; 2020. Accessed October 12, 2022. https://www.who.int/teams/immunization-vaccines-and-biologicals/strategies/global-vaccine-action-plan

[10] Every Woman, Every Child. *The Global Strategy for Women’s Children’s and Adolescents’ Health (2016-2030)*. United Nations; 2015. Accessed October 12, 2022. https://data.unicef.org/resources/global-strategy-womens-childrens-adolescents-health/

[11] World Health Organization (WHO). *Global Routine Immunization Strategies and Practices (GRISP): A Companion Document to the Global Vaccine Action Plan (GVAP)*. WHO; 2016. Accessed October 12, 2022. https://apps.who.int/iris/handle/10665/204500

[12] Gavi, the Vaccine Alliance. *Equity from Birth: An Integrated Approach to Immunization and Nutrition Policy Brief*. Gavi; 2021. Accessed October 12, 2022. https://scalingupnutrition.org/wp-content/uploads/2021/10/Gavi_Policy-Brief-Gavi-SUN_En.pdf

[13] Chehab ET Anya BPM Onyango AW. Experience of integrating vitamin A supplementation into polio campaigns in the African Region. Vaccine. 2016;34(43):5199–5202. 10.1016/j.vaccine.2016.05.056. 27364094

[14] Mihigo R Anya B Okeibunor J. African vaccination week as a vehicle for integrated health service delivery. BMC Health Serv Res. 2015;15(1):358. 10.1186/s12913-015-0989-7. 26328630 PMC4557633

[15] Hodges MH Sesay FF Kamara HI. Integrating vitamin A supplementation at 6 months into the Expanded Program of Immunization in Sierra Leone. Matern Child Health J. 2015;19(9):1985–1992. 10.1007/s10995-015-1706-1. 25665894 PMC4521092

[16] World Health Organization (WHO). Regional Office for Africa. *Reaching Every District (RED): A Guide to Increasing Coverage and Equity in All Communities in the African Region*. WHO; 2017. https://www.afro.who.int/sites/default/files/2018-02/Feb%202018_Reaching%20Every%20District%20%28RED%29%20English%20F%20web%20v3.pdf

[17] Central Statistical Authority/Ethiopia (CSA) and ORC Macro. *Ethiopia Demographic and Health Survey, 2000*. CSA/Ethiopia and ORC Macro; 2001. Accessed October 12, 2022. https://dhsprogram.com/publications/publication-fr118-dhs-final-reports.cfm

[18] Central Statistical Authority/Ethiopia (CSA) and ORC Macro. *Ethiopia Demographic and Health Survey, 2016*. CSA/Ethiopia and ORC Macro; 2016. Accessed October 12, 2022. https://dhsprogram.com/publications/publication-FR328-DHS-Final-Reports.cfm

[19] Ethiopia Ministry of Health (MOH). *Health Sector Transformation Plan II (HSTP II): 2020/21-2024/25 (2013 EFY - 2017 EFY)*. MOH; 2021. Accessed October 12, 2022. https://www.familyplanning2020.org/sites/default/files/HSTP-II.pdf

[20] Central Statistical Authority/Ethiopia (CSA) and ORC Macro. *Ethiopia Demographic and Health Survey, 2011*. CSA/Ethiopia and ORC Macro; 2012. Accessed October 12, 2022. https://dhsprogram.com/publications/publication-fr255-dhs-final-reports.cfm

[21] Tadesse AW Aychiluhm SB Mare KU. Individual and community-level determinants of iron-folic acid intake for the recommended period among pregnant women in Ethiopia: a multilevel analysis. Heliyon. 2021;7(7):e07521. 10.1016/j.heliyon.2021.e07521. 34296017 PMC8282952

[22] World Health Organization (WHO). *Guideline: Counselling of Women to Improve Breastfeeding Practices*. WHO; 2018. Accessed October 12, 2022. https://www.who.int/publications-detail-redirect/9789241550468 30933442

[23] World Health Organization (WHO). *WHO Recommendations on Antenatal Care for a Positive Pregnancy Experience*. WHO; 2016. Accessed October 12, 2022. https://www.who.int/publications-detail-redirect/9789241549912 28079998

[24] World Health Organization (WHO). *Complementary Feeding: Report of the Global Consultation, and Summary of Guiding Principles for Complementary Feeding of the Breastfed Child*. WHO; 2003. Accessed October 12, 2022. https://apps.who.int/iris/handle/10665/42739

[25] World Health Organization (WHO). *Global Strategy for Infant and Young Child Feeding*. WHO; 2003. Accessed October 12, 2022. https://www.who.int/publications-detail-redirect/9241562218

[26] Federal Democratic Republic of Ethiopia. *National Nutrition Program 2016-2020*. National Nutrition Coordinating Body; 2016. https://extranet.who.int/nutrition/gina/sites/default/filesstore/ETH%202016%20National%20Nutrition%20Programme%20II.pdf

[27] Maternal and Child Survival Program (MCSP). *Addressing the Denominator Conundrum for Maternal and Child Health Programs: A New Methodology*. MCSP; 2019. https://publications.jsi.com/JSIInternet/Inc/Common/_download_pub.cfm?id=22149&lid=3

[28] Umeh GC Madubu DM Korir C. Micro-planning for immunization in Kaduna State, Nigeria: lessons learnt, 2017. Vaccine. 2018;36(48):7361–7368. 10.1016/j.vaccine.2018.10.020. 30366806 PMC6238078

[29] Cooper CM Fields R Mazzeo CI. Successful proof of concept of family planning and immunization integration in Liberia. Glob Health Sci Pract. 2015;3(1):71–84. 10.9745/GHSP-D-14-00156. 25745121 PMC4356276

[30] Nelson AR Cooper CM Kamara S. Operationalizing integrated immunization and family planning services in rural Liberia: lessons learned from evaluating service quality and utilization. Glob Health Sci Pract. 2019;7(3):418–434. 10.9745/GHSP-D-19-00012. 31558598 PMC6816810

[31] Wallace A Ryman T Mihigo R. Strengthening evidence-based planning of integrated health service delivery through local measures of health intervention delivery times. J Infect Dis. 2012;205(Suppl 1):S40–S48. 10.1093/infdis/jir775. 22315385

[32] Huntington D Aplogan A. The integration of family planning and childhood immunization services in Togo. Stud Fam Plann. 1994;25(3):176–183. 10.2307/2137943. 7940622

[33] Ryman TK Wallace A Mihigo R. (2012). Community and health worker perceptions and preferences regarding integration of other health services with routine vaccinations: four case studies. J Infect Dis. 2012; 205 Suppl 1:S49–S55. 10.1093/infdis/jir796. 22315386

[34] Aguayo VM Baker SK Crespin X Hamani H MamadoulTaïbou A. Maintaining high vitamin A supplementation coverage in children: lessons from Niger. Food Nutr Bull. 2005;26(1):26–31. 10.1177/156482650502600103. 15810796

[35] Bwalya M. *Zambia National Measles Immunization Campaign 2003: Technical Report*. Zambia Ministry of Health; 2003.

[36] Salam RA Das JK Bhutta ZA. Integrating nutrition into health systems: what the evidence advocates. Matern Child Nutr. 2019;15(Suppl 1):e12738. 10.1111/mcn.12738. 30748112 PMC6594109

[37] Malaria vaccine: WHO position paper-March 2022. *Wkly Epidemiol Rec*. 2022;97(9):61–80. Accessed October 24, 2022. https://www.who.int/publications/i/item/who-wer9709-61%E2%80%9380

